# What we learned from lifting COVID-19 restrictions in Macao in December 2022

**DOI:** 10.7150/ijbs.86875

**Published:** 2023-10-24

**Authors:** Chon Lok LEI, Hoi Man NG, Guihui QIN, Cheung Kwan YEUNG, Chon Leng LEI, Ren-He XU

**Affiliations:** 1 Faculty of Health Sciences, University of Macau, Macao, China.; 2 Kiang Wu Hospital, Macao, China.

The World Health Organization has recently declared that the coronavirus disease 2019 (COVID-19) no longer constitutes a public health emergancy of international concern. During the past three years, severe acute respiratory syndrome coronavirus 2 (SARS-CoV-2) that caused the pandemic of the disease evolved through multiple variants from Alpha to Beta, Gamma, Delta, and Omicron, a milder variant 1-3. Long COVID has also been reported, referring to symptoms that last for several weeks or months beyond the initial illness 4. Vaccination, including both the urgently developed mRNA vaccines (RV) and the conventional inactivated viral vaccines (IV), proved effective to reduce the severity and death rate of the disease 5, 6. However, its necessity and efficacy, if any, to prevent COVID-19 caused by *mild* viral variants such as Omicron remains to be evaluated as it is critical for justification of future health policy.

To achieve this goal, we conducted a survey in Macao between December 2022 and January 2023 when Macao was hit by an outbreak of the Omicron. Macao, a special administrative region of China with a population of around 683 thousand, took intensive non-pharmaceutical measures for public health and social interventions to control the local outbreaks of COVID-19 in the tightly bordered territory. It successfully kept the total number of local infections under hundreds of cases before June 2022. Although the number rose to thousands between June to August, but quickly dropped down to zero for local cases and maintained so afterward 7. Thus, Macao successfully avoided heavy impact by the more severe variants including Alpha, Beta, Gamma, and Delta. However, an outbreak occurred in Macao in December 2022 when the milder variant Omicron became dominant and Macao lifted most of its COVID-19 restrictions and reopened its borders. By then, the vaccination coverage of the entire population with the Sinopharm inactivated viral vaccine (IV), Pfizer-BioNTech mRNA vaccine (RV), or their heterologous combinations was around 93% for ≥ 2 doses and 57% for ≥ 3 doses 7. Therefore, Macao is an ideal region for conducting research on the efficacy of different types of vaccines against the Omicron variant without the interference caused by other SARS-CoV-2 variants 8. We received 626 replies from the survey, among 345 participants who had SARS-CoV-2-positive tests (via either rapid antigen test or nucleic acid test) and were all at the age of 18-64 except 15 at 65 or over (the survey did not require to provide exact age).

The statistical results show that 18% (61 out of 345) of the responders reported no symptoms and the rest complained only mild symptoms, which mainly lasted less than 7 days, including fever (mostly at 38-38.9°C of the body temperature) and various degrees of cough, throat pain, and fatigue (Figure 1A). None of the responders reported hospitalization and ventilator usage. Thus, they can be classified as “no/mild symptoms”. We performed a Bayesian analysis for these rating data with an ordered-probit model 9 (Figure 1B) and found no strong difference between the means of the severity and duration among the different vaccination groups (Figure 1C). More strikingly there is no significant difference in the severity and duration of the symptoms between those with and without vaccinations. Unfortunately, we do not have information about prior SARS-CoV-2 infection of the participants in the survey which could lead to a milder version of second infection, potentially biasing the results. Nonetheless, the overall rate of prior SARS-CoV-2 infections was very low (less than 0.3%) in Macao before December 2022, therefore it should not remarkably affect these results.

It is also worth noting that our data are derived from a rather small number of participants mostly at young/middle ages including a low percentage of non-vaccinated individuals, which could also cause some biases to our results. Many previous studies (*e.g.* those in Hong Kong) have addressed similar issues on severe cases and death rates 10. Thus, our findings still provide a valuable view that perhaps no vaccination is needed to prevent *mild* COVID-19 such as that caused by Omicron. Further study is necessary to better understand the effectiveness and safety of the vaccines on mild variants. We propose to seriously complete long-term safety studies of the new vaccine types such as RV that was developed in a rush 6. We also hope that this short study stimulates more discussions on the safety and effectiveness of COVID-19 vaccines.

## Figures and Tables

**Figure 1:**
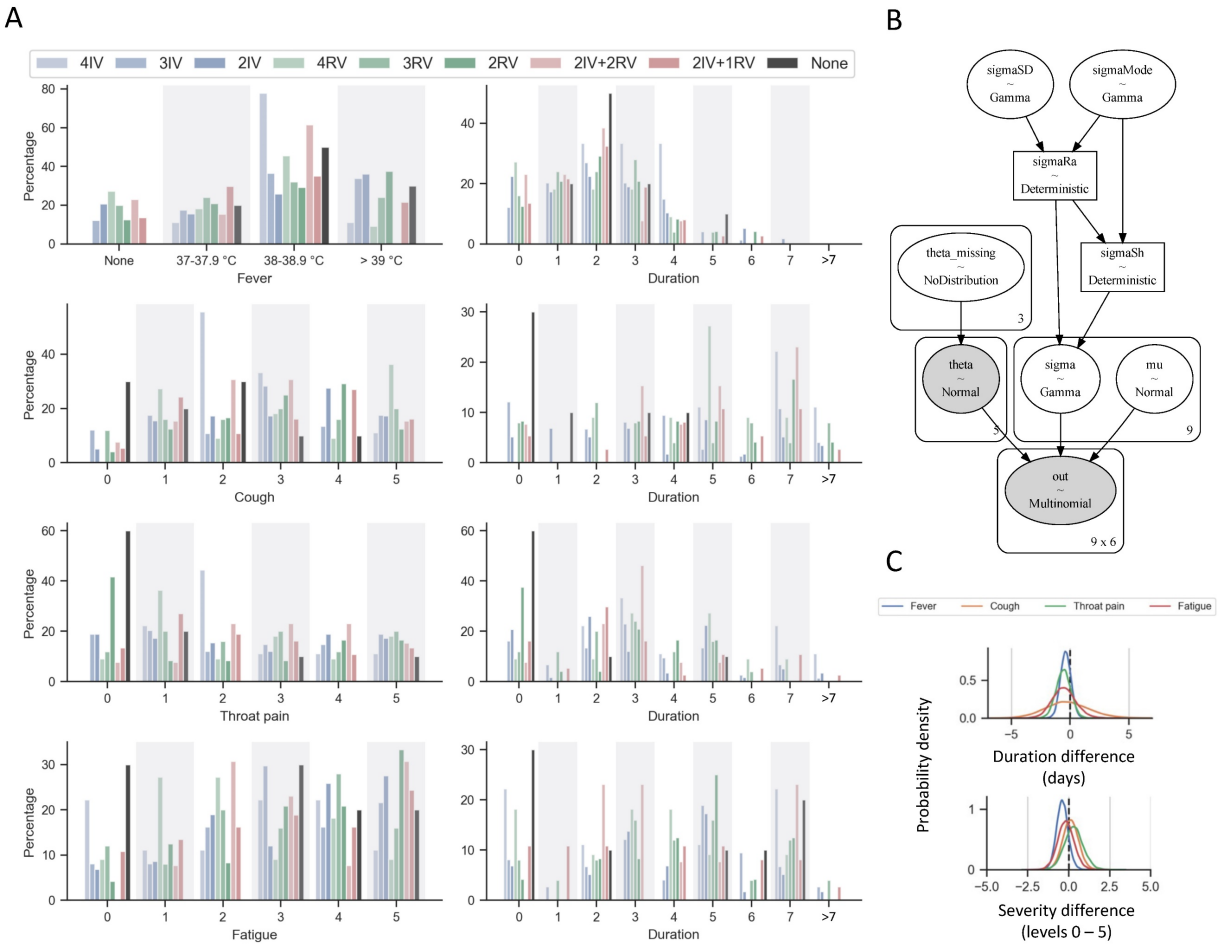
** Results of self-reported survey of symptom responses to COVID-19 infection in Macao during December 2022 to January 2023.** (A) Responses were split by vaccination groups or no vaccination history prior to infection. The numbers of subject were *N* = 9, 74, 58, 11, 25, 24, 13, 37, 10 for 4IV, 3IV, 2IV, 4RV, 3RV, 2RV, 2IV+2RV, 2IV+1RV, and none, respectively. The bar charts represent percentage of responses *within* each group. For symptoms, cough, throat pain, and fatigue, 0 indicates no such a symptom to 5 being extreme severe. (B) Schematic overview of the Bayesian model for the survey response analysis using an ordered-probit model. (C) Typical statistical results showing the probability densities of the mean duration differences (days) and the mean severity response differences (levels 0-5) between 3RV and 3IV. Other pairwise comparions show similar results.
